# Why do patients still die from gestational trophoblastic neoplasia? System failures beyond curability

**DOI:** 10.1590/1806-9282.20260522

**Published:** 2026-08-28

**Authors:** Antonio Braga, Sue Yazaki Sun, Edward Araujo, Jorge de Rezende-Filho

**Affiliations:** 1 Universidade Federal do Rio de Janeiro, School of Medicine, Department of Gynecology and Obstetrics – Rio de Janeiro (RJ), Brazil.; 2 Universidade Federal do Estado do Rio de Janeiro, School of Medicine and Surgery, Department of General and Specialized Surgery – Rio de Janeiro (RJ), Brazil.; 3 Universidade de Vassouras, Postgraduate Program in Applied Health Sciences – Vassouras (RJ), Brazil.; 4 Universidade Federal de São Paulo, Paulista School of Medicine, Department of Obstetrics – São Paulo (SP), Brazil.; 5 Universidade Municipal de São Caetano do Sul, Discipline of Woman Health – São Caetano do Sul (SP), Brazil.

Gestational trophoblastic neoplasia (GTN) has been classically described as “God’s first cancer and man’s first cure,” a phrase commonly attributed to Arthur Hertig, reflecting both its origin from placental tissue and its historic role as the first disseminated malignancy to be cured with chemotherapy^1^. It stands as a unique entity in oncology – one of the most curable malignancies and a landmark success of modern chemotherapy^2^. Yet, despite this, deaths from GTN still occur^3^. This contradiction, remarkable curability coexisting with persistent mortality, demands careful reflection.

GTN comprises a spectrum of malignant disorders arising from trophoblastic tissue, characterized by abnormal proliferation and the ability to metastasize^4^. Approximately half of GTN cases develop after a molar pregnancy^5^. However, the remaining cases follow non-molar gestations, including term or preterm deliveries, miscarriages, and ectopic pregnancies. This latter group poses a particular diagnostic challenge, as the antecedent pregnancy is not typically regarded as a sentinel event, and the subsequent presentation, frequently marked by abnormal vaginal bleeding and, in some cases, metastatic disease without an identifiable primary site, may obscure the diagnosis of GTN^6^.

Despite its malignant nature, GTN is a rare disease. This rarity justifies, and indeed mandates, its management in specialized reference centers, where expertise is concentrated, and outcomes are optimized. Centralization has consistently been associated with reduced mortality^3^. However, it introduces a paradox: while concentrating expertise improves outcomes, it may also contribute to delayed recognition in non-specialized settings^7^. Many clinicians will encounter few, if any, cases throughout their careers. This reinforces the need to strengthen education on GTN at both undergraduate and postgraduate ­levels, ensuring that early warning signs are recognized promptly.

Another distinctive aspect of GTN is that, although it is a cancer, its management is not confined to a single specialty. In Brazil, treatment is often led by obstetricians; in the United States, by gynecologic oncologists; and in the United Kingdom, frequently by medical oncologists within centralized systems. This variability underscores an essential principle: optimal care depends less on specialty labels and more on access to experienced teams. Clinicians must be aware of where expertise is concentrated within their healthcare system to ensure timely referral^8^.

A critical and often underappreciated issue in GTN management is the misconception that histopathological confirmation is required before initiating treatment. Unlike most malignancies, GTN is primarily diagnosed biochemically^6^. The hallmark of the disease is an abnormal human chorionic gonadotropin (hCG) profile – plateauing or rising levels after a pregnancy event. Waiting for histological confirmation not only delays treatment but may expose patients to unnecessary and potentially dangerous procedures. Biopsies of metastatic lesions, often highly vascular, carry a significant risk of hemorrhage and should be avoided. In GTN, prompt recognition of abnormal hCG kinetics is more important than tissue diagnosis^6^.

The same principle of “less is more” applies to staging. In approximately 80% of cases, the disease is confined to the uterus. A careful clinical evaluation, transvaginal pelvic ultrasound with Doppler, and a chest X-ray are sufficient for initial staging in most patients^6^. The routine use of computed tomography, particularly chest CT, may detect micrometastases that do not alter prognosis but can lead to overtreatment^9^. Moreover, ­reliance on advanced imaging may delay treatment initiation, especially in resource-limited settings. In GTN, timeliness is critical, and excessive staging may be counterproductive.

Once staging is completed, management decisions must be individualized. In non-metastatic disease, two strategies deserve consideration. Primary hysterectomy may be appropriate for women with completed childbearing, particularly in older patients, reducing the need for chemotherapy^10^. Alternatively, a second uterine evacuation has been proposed as a means of reducing tumor burden and, in selected cases, avoiding chemotherapy^11^. While evidence supporting this approach remains debated, ongoing studies continue to refine its role. These options highlight the importance of tailoring treatment to patient characteristics and preferences.

Risk stratification using the World Health Organization (WHO)/International Federation of Gyneclogy and Obstetrics (WHO/FIGO) scoring system remains central to management^6^. This system identifies patients at higher risk of chemoresistance who require multiagent chemotherapy from the outset^12^. For high-risk GTN, the EMA/CO regimen (containing etoposide, methotrexate, actinomycin-D, alternating weekly with cyclophosphamide and vincristine [oncovin]) remains the standard of care, achieving remarkable cure rates even in advanced, metastatic, or previously treated disease^13^. Conversely, initiating treatment with single-agent chemotherapy in high-risk patients increases the likelihood of treatment failure and may contribute to mortality^3^.

Another key determinant of outcome is the timely recognition of chemoresistance. GTN is one of the few cancers in which a reliable tumor marker – hCG – allows real-time monitoring of treatment response^14^. Failure to recognize plateauing or rising hCG levels can delay escalation of therapy, compromising outcomes. For this reason, strict adherence to monitoring protocols is essential. Maintaining the reliability of hCG measurement also requires effective contraception during follow-up, as a new pregnancy would confound interpretation^15^.

In patients with extensive disease burden or critical clinical presentation, special therapeutic considerations are required^1,6^. Ultra-high-risk GTN is associated with a significant risk of early mortality, particularly within the first weeks of treatment. In such cases, induction with low-dose etoposide and cisplatin (EP) before full-dose combination chemotherapy reduces early deaths by mitigating tumor lysis and hemorrhagic complications^16^. Furthermore, platinum-based regimens, such as EP/EMA, consisting of etoposide and cisplatin (EP) alternating weekly with etoposide, methotrexate, and actinomycin D (EMA) or TP/TE, consisting of paclitaxel plus cisplatin (TP) alternating weekly with paclitaxel plus etoposide (TE), should be employed in ultra-high-risk or refractory cases, reflecting the need for aggressive but carefully staged treatment^6^.

More recently, immunotherapy has emerged as a promising addition to the therapeutic arsenal^17,18^. The use of immune checkpoint inhibitors, particularly pembrolizumab, is supported by a strong biological rationale, given the high expression of PD-L1 in trophoblastic tumors. Clinical data have demonstrated meaningful responses in patients with refractory GTN, offering a new line of therapy in otherwise resistant disease. The incorporation of immunotherapy represents a significant advance and may redefine the management of selected patients in the coming years^1^.

Notwithstanding the occurrence of these deaths, many cases of GTN–related mortality remain largely invisible within conventional maternal mortality indicators. Although deaths from GTN are unequivocally classified as direct obstetric deaths, their temporal distribution frequently leads to systematic underrecognition. By definition, maternal death includes those occurring during pregnancy or within 42 days of its termination (ICD-10: O00–O99). However, a substantial proportion of GTN-related deaths occur beyond this period, being classified as late maternal deaths (between 42 days and 12 months after the end of pregnancy; ICD-10: O96) and, not infrequently, as remote maternal deaths (occurring more than 12 months after pregnancy; ICD-10: O97).

Because fatal GTN may evolve insidiously, be diagnosed late, or follow a prolonged, chemoresistant course, death commonly occurs well beyond the conventional 42-day window. As a consequence, many of these deaths are not captured in routine maternal mortality statistics, are underrepresented in the analyses conducted by maternal mortality committees, and receive limited attention from health system managers. This represents a critical gap in surveillance, leading to underestimation of the true burden of GTN and weakening system-level accountability.

Recognizing GTN-related deaths across the full temporal spectrum of maternal mortality is essential to accurately characterize disease impact and to inform public health strategies. Failure to do so perpetuates the paradox of preventable deaths in a highly curable disease, not as a consequence of therapeutic limitations, but of systemic invisibility.

After all, if GTN is so curable, why do patients still die?

The answer may lie not solely in the biology of the disease, but also in the organization of care^8^. Delayed diagnosis, inappropriate initial management, inadequate risk stratification, and lack of timely referral to specialized centers remain the main drivers of mortality^3,4^. Studies consistently show that treatment outside reference centers is associated with worse outcomes, underscoring access to specialized care as the most important modifiable determinant of mortality^3,19^.

In countries with large geographic dimensions, such as Brazil, distance from reference centers has also been identified as a determinant of outcome. Patients living far from specialized units may face delays in diagnosis, referral, and treatment initiation^20^. Addressing this challenge requires innovative solutions. Telemedicine, telemonitoring, and structured referral networks can help bridge geographic gaps, allowing expertise to extend beyond physical boundaries^21^.

The Brazilian experience in organizing care for GTN demonstrates that centralization, when combined with coordinated networks, can significantly improve outcomes^8^. This model offers valuable lessons for other regions facing similar challenges.

Ultimately, deaths from GTN are largely preventable. They reflect gaps in recognition, access, and organization of care. Bridging these gaps requires not only scientific knowledge but also system-level commitment. Because in GTN, cure is not the exception – it should be the rule.

## Data Availability

The datasets generated and/or analyzed during the current study are available from the corresponding author upon reasonable request.
